# Investigating the Durability of Iodine Waste Forms in Dilute Conditions

**DOI:** 10.3390/ma12050686

**Published:** 2019-02-26

**Authors:** R. Matthew Asmussen, Joseph V. Ryan, Josef Matyas, Jarrod V. Crum, Joelle T. Reiser, Nancy Avalos, Erin M. McElroy, Amanda R. Lawter, Nathan C. Canfield

**Affiliations:** Energy and Environment Directorate, Pacific Northwest National Laboratory, Richland, WA 99352, USA; joe.ryan@pnnl.gov (J.V.R.); josef.matyas@pnnl.gov (J.M.); jarrod.crum@pnnl.gov (J.V.C.); Joelle.t.reiser@pnnl.gov (J.T.R.); Nancy.avalos@pnnl.gov (N.A.); erin.mcelroy@wsu.edu (E.M.M.); amanda.lawter@pnnl.gov (A.R.L.); nathan.canfield@pnnl.gov (N.C.C.)

**Keywords:** iodine, waste form, corrosion, microscopy, silver iodide

## Abstract

To prevent the release of radioiodine during the reprocessing of used nuclear fuel or in the management of other wastes, many technologies have been developed for iodine capture. The capture is only part of the challenge as a durable waste form is required to ensure safe disposal of the radioiodine. This work presents the first durability studies in dilute conditions of two AgI-containing waste forms: hot-isostatically pressed silver mordenite (AgZ) and spark plasma sintered silver-functionalized silica aerogel (SFA) iodine waste forms (IWF). Using the single-pass flow-through (SPFT) test method, the dissolution rates respective to Si, Al, Ag and I were measured for variants of the IWFs. By combining solution and solid analysis information on the corrosion mechanism neutral-to-alkaline conditions was elucidated. The AgZ samples were observed to have corrosion preferentially occur at secondary phases with higher Al and alkali content. These phases contained a lower proportion of I compared with the matrix. The SFA samples experienced a higher extent of corrosion at Si-rich particles, but an increased addition of Si to the waste led to an improvement in corrosion resistance. The dissolution rates for the IWF types are of similar magnitude to other Si-based waste form materials measured using SPFT.

## 1. Introduction

In the reprocessing of used nuclear fuel, radioiodine will be released, primarily during the dissolution of the fuel [1]. A portion of this iodine is ^129^I with a half-life of 15.7 million years; to prevent discharge of this long-lived radionuclide, the released iodine needs to be captured in the off-gas management system of the reprocessing facility. Multiple approaches to removing the iodine from the off-gas system (which contains large amounts of water and NO_X_) and can be grouped into: (A) wet scrubbing methods such as Mercurex, Iodox, electrolytic scrubbing, and alkaline scrubbing [2,3]; and (B) solid sorbent capture including resins [4], carbon-based materials [5,6,7], metal organic frameworks [8,9], zeolites [10,11,12], silica [13] and aerogels [14,15,16]. The wet scrubbing processes would all require a secondary process(es) for the iodine-loaded product to be converted to a waste form such as grouting or vitrification. One of the primary advantages of solid sorbents is their potential to be readily transformed into a final waste form, through either direct post-processing in a canister or densification.

The presence of silver (Ag) in solid sorbents can enhance iodine capture through the generation of silver iodide (AgI) in the material. AgI is widely considered of as a desirable form of iodine for disposal because it has a low solubility (AgI K_sp_ = 8 × 10^−17^) [17]. However, the stability of AgI can be impacted by its local environment, as its dissolution can be highly affected by redox conditions [18], increasing pH or the presence of sulfide [19]. Placing the AgI within a durable matrix can ensure further protection in long-term disposal.

While many solid sorbents with and without Ag have been developed for iodine capture, the consolidated waste form development for the materials and their associated durabilities have been sparsely investigated. The durabilities of these candidate waste forms need to be understood to facilitate predictions of their behavior over long disposal time frames in a repository.

Two of the most technologically mature iodine waste forms (IWF) developed to date are silver-exchanged zeolites, such as silver mordenite (AgZ) [20,21,22], and silver-functionalized silica aerogels (SFA) [14,23,24]. Silver exchanged zeolites, specifically the mordenite form that has higher Si:Al ratio compared with faujasite zeolites [25], have been researched for iodine capture in the US. Reduced Ag (Ag^0^) is present in the AgZ to reduce iodine (I_2_) to iodide (I^−^) and the eventual formation of AgI while in use [20]. The loaded AgZ can then be converted to a final waste form through post-processing in a canister [22,26]. These demonstrations have been performed using hot isostatic pressing (HIP) and hot uniaxial pressing (HUP) to create a consolidated AgZ in steel canisters. 

SFAs have been developed in the last decade as a moderate specific surface area (~150 m^2^/g) material for iodine capture. An aerogel backbone can be thiolated and functionalized with Ag^0^ nanoparticles to create the SFAs [23]. The SFAs are capable of high iodine loadings (up to 40 wt %), are stable in expected off-gas operating conditions (e.g., high humidity and NO_x_) and can be directly densified to a final waste form using HIP or spark plasma sintering (SPS). In both processes, the application of heat collapses the aerogel backbone, reducing its volume and eliminating void spaces to create a final, high density waste form. This densification process has been demonstrated previously [23].

This work presents the first study of the corrosion behavior of AgZ and SFA based IWF in aqueous environments using the single-pass flow-through (SPFT) technique [27]. The consolidated AgZ and densified SFA samples are comprised of multi-component microstructures, which may lead to heterogeneous dissolution of the waste form. To assess such behavior, monolithic samples of each material were evaluated to track corrosion using solution and solid analyses. The materials were evaluated pre- and post-corrosion with optical microscopy, optical profilometry, electron microscopy, and X-ray diffraction (XRD). This study will help inform further development of IWF to improve durability and the data within can be used to develop long-term predictive models for iodine releases from candidate waste forms. It should be made clear that the IWF samples used in this study have not yet been optimized for durability, but can be used as comparisons for any future assessments of IWF durability.

## 2. Materials and Methods 

### 2.1. Materials

**Figure 1:**
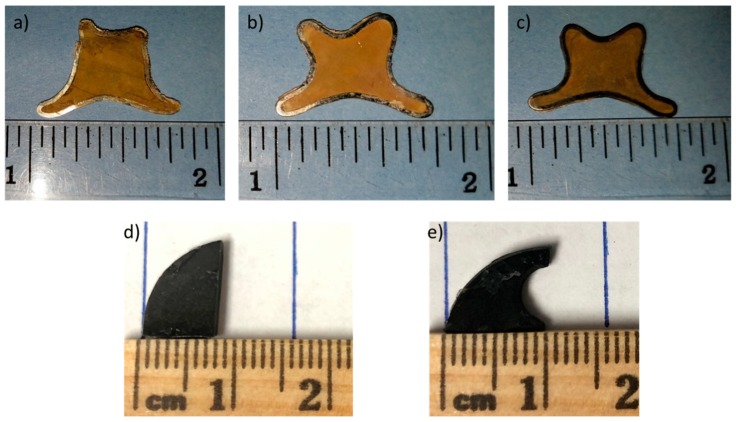
Photographs showing the samples used in this study: (**a**) AgZ 1-3; (**b**) AgZ 1-7; (**c**) AgZ 1-8; (**d**) SPS-1; and (**e**) SPS-2.

The AgZ samples were prepared at Oak Ridge National Laboratory [26]. The base zeolite used was Ionex Type Ag 900 E16 from Molecular Products and had a chemical composition of Ca_8_(Al_8_Si_40_O_96_)∙24H_2_O [22] with ~9 wt % Ag content. A steel cylindrical canister (25 mm” diameter, 75 mm” tall) was filled with AgZ and HIPed for 3 h at 175 MPa. Three samples were included in this testing: AgZ 1-3 (HIPed at 700 °C at 175 MPa for 3 h, no iodine loading), AgZ 1-7 (HIPed at 525 °C at 175 MPa for 3 h, loaded with iodine) and AgZ 1-8 (HIPed at 700 °C at 175 MPa for 3 h, loaded with iodine); the numeration sequence corresponds to the previously reported sample preparation [28]. From the canisters, two horizontal pucks were sectioned from the ingot (2 mm thick) to produce a flat surface encapsulated in a steel ring. Visual images of the samples are shown in Figure 1a–c. The chemical makeup of the AgZ samples was determined using energy dispersive X-ray spectroscopy (EDS, Bruker Quantax 6|60; Bruker Nano GmbH, Berlin, Germany) by taking the average composition of a minimum of four 250 µm × 350 µm areas on the sample and these are listed in Table 1. Because there were only two small AgZ samples, and to keep the integrity of the samples, no digestion for chemical composition was conducted. 

The spark plasma sintered (SPS) SFA samples were prepared using SFA fabricated at Pacific Northwest National Laboratory using a commercially available silica aerogel from United Nuclear (Laingbrugh, MI, USA). The as-received SFA materials were functionalized in-house using the method reported previously [29]. Two samples were used in the study and given the designations of SPS-1 and SPS-2. SPS-1 was densified without alterations to the materials while the SPS-2 sample included an additional 20 wt % of raw SFA added prior to the SPS process. The samples (~5 g) were sintered in a graphite die set and heated to 1200 °C (ramp of 100 °C/min) under Ar atmosphere. The temperature was held at 1200 °C for 30 min at 70 MPa and allowed to cool to room temperature under Ar atmosphere until below 400 °C. The final waste form samples to be used in the testing are shown in Figure 1d,e. Because there was only a single sample for each condition, no digestion for chemical composition was possible. The compositions of the SPS-SFA samples were determined from the original SFA material prior to densification and are given in Table 2. The densified SFA materials are highly sensitive to electron beam exposure and lose I with increased exposure time. A difference between the composition measured with EDS for SFA and the actual composition has been observed in a previous study [23]. Thus, EDS compositions were not used for the SFA in this work.

One face of the each sample was polished prior to being exposed to the SPFT test. For the AgZ samples, the faces polished for each of the samples were adjacent to each other when cut. The samples were placed on a rotating polishing unit at 15 µm SiC for 20 min with 20 lbs of force at 240 rpm, followed by successive 5 min sets at 9 µm and 3 µm. Following these steps, the samples were finalized on a vibratory polisher with 1-µm SiC followed by 0.05-µm colloidal silica for 4 h each. A final ethanol rinse was used to remove any remaining debris. The opposite face of the samples was then masked using room temperature vulcanizing (RTV) silicone (Locktite®). The masking was done to limit damage to the samples as there were only a few unique samples.

### 2.2. Single-Pass Flow-Through Testing

Corrosion testing of the samples was performed with the SPFT technique following ASTM Method C1662-17 [27]. In general, the SPFT test utilizes a flowing solution through a saturated, sealed vessel containing a sample and the effluent solution from the reactor was monitored. The monolithic samples were placed on a cage within a 60 mL reaction vessel made of high-density polyethylene (HDPE, Savillex) with the polished face directed upward. The flow rate was provided by a syringe pump (Nordgren-Khloen, V6 syringe drive pump, Las Vegas, NV, USA) and was targeted at 20 mL/day to provide dilute conditions (<5 mg/L for species in solution) yet keep the concentrations within a measurable range (above instrument detection limits). All experiments were performed in an oven, in open atmosphere (sealed reactor), at 90 °C. Effluent samples were collected in polytetrafluorethylene (PTFE) bottles and flow rates were determined gravimetrically. Only a single test was performed at pH 7 and pH 11 to preserve the unique samples due to the loss of material experienced during the test and the post-test polishing procedure. A repeat experiment at shorter duration (17 days compared to 36 days) was performed on the AgZ samples at pH 9 to ensure reproducibility of the SPFT technique. 

At the conclusion of the test, flow to the reactors was stopped and the samples were removed and rinsed three times with double deionized water (18.2 MΩ·cm) and three times with anhydrous ethanol (98%, Fisher Scientific). Solutions buffered at pH (at room temperature, RT) 7 and pH 9 were made with 0.05 M tris(hydroxylmethyl)aminomethane (TRIS, Fisher Scientific) adjusted to the desired pH using HNO_3_, while solutions at pH (RT) 11 were a 0.001 M LiOH + 0.01 M LiCl solution. 

### 2.3. Post Analysis

Concentrations of the analytes in the collected effluents were measured using inductively coupled plasma (ICP) mass spectroscopy (Thermo X-Series 2, Waltham, MA, USA) for total I (detection limit of 1.26 µg/L) and ICP optical emission spectroscopy (Perkin Elmer Optima 8300 DV, Perkin Elmer, Shelton, CT, USA) for Si, Ag and Al (with detection limits of 54.6 µg/L, 17.9 µg/L and 15.6 µg/L, respectively). 

The sample surfaces were imaged using scanning electron microscopy (SEM) and elemental distributions were determined using EDS. Attempts were made to correlate the same area on the sample surface both before and after corrosion. Images were collected at 70× and 250× magnifications. SEM analyses were performed with a JSM-7001F microscope (JEOL USA, Inc., Peabody, MA, USA) with an XFlash 6|60 EDS Si-drift detector (Bruker) for elemental mapping and spot analysis.

The samples were also characterized post-corrosion for any changes in their structure using X-ray diffraction (XRD). The samples were not altered prior to the XRD measurements; they were analyzed as intact coupons. The XRD patterns were collected with a Bruker D8 Advance XRD system (Bruker AXS, Tuscon, AZ, USA) equipped with a Cu target (Kα1 = 0.15406 nm) over a scan range of 5° 2θ to 75° 2θ using a step size of 0.015° 2θ and a hold time of 4 s per step. The scans were analyzed with TOPAS (v4.2) whole pattern fitting software according to the fundamental parameters approach [30]. Structure patterns were selected from the Inorganic Crystal Structure Database (release 2013) with unit cell dimensions refined in the fitting process of each pattern. 

The topographical evolution of the surface following corrosion was observed using optical profilometry (OP) on a Bruker GTK profilometer with a 5× or 50× lens before and after corrosion. 

## 3. Results

### 3.1. Pre-Corrosion Characterization 

**Figure 2:**
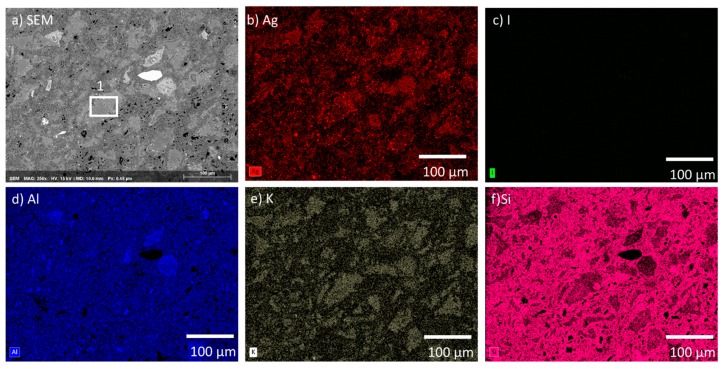
Microstructure of the AgZ 1-3 sample shown by: (**a**) SEM micrograph; and the corresponding EDS maps of: (**b**) Ag; (**c**) I; (**d**) Al; (**e**) K; and (**f**) Si. The large white inclusion in (**a**) is made of Zr and S whose origin are not known. The white box marked “1” is the location where an EDS spot analysis was performed and listed in Table 3.

The initial microstructures of the AgZ samples prior to corrosion were observed with SEM and EDS. The multiphase structure of AgZ 1-3 is shown in the SEM micrograph in Figure 2a. The microstructure contained a continuous matrix and large isolated secondary phases (the lighter grey regions in the SEM image) within the matrix. The Ag particles (Figure 2b) were present in both the matrix and in the secondary phases. No iodine was present in AgZ 1-3 (Figure 2c). The secondary phases present within the matrix contained high amounts of Al and K (i.e., Figure 2d,e, respectively). Si comprised the matrix phase (Figure 2f). The larger white inclusion in the center of Figure 2a was composed primarily of Zr and S, the origin of which is unknown. An example of a commonly observed secondary phase is highlighted with a white box in Figure 2a. EDS analysis of this location, shown in Table 3, revealed higher amounts of K (1.5 wt %) than the matrix as a whole. This phase also contained Ag inclusions.

**Figure 3:**
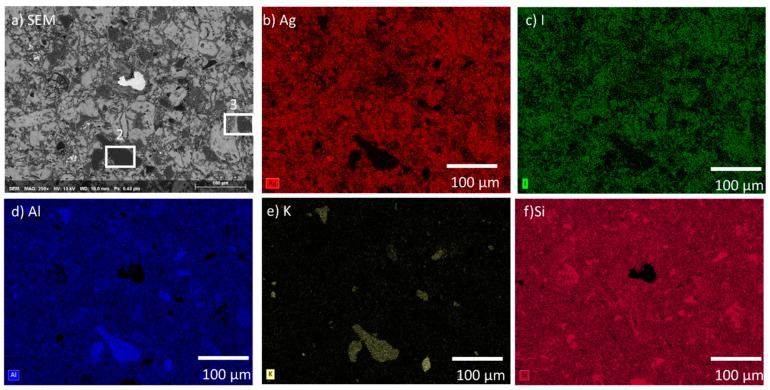
Microstructure of the AgZ 1-7 sample shown by: (**a**) SEM micrograph; and the corresponding EDS maps of: (**b**) Ag; (**c**) I; (**d**) Al; (**e**) K; and (**f**) Si. The large white inclusion in (**a**) is comprised of Fe and Mn whose sources are not known. The white boxes marked “2 and 3” are the locations where an EDS spot analysis was performed and listed in Table 3.

The AgZ 1-7 sample is shown in Figure 3a. AgZ 1-7 had similar features to AgZ 1-3. The two main differences between AgZ 1-3 and AgZ 1-7 were the presence of I (Figure 3c), and more even distribution of Ag in the AgZ 1-7 sample. The I and Ag distribution in the AgZ 1-7 sample were observed to be even with one another and with few discrete Ag particles, such as those observed in the AgZ 1-3 sample. These changes were possibly due to the higher temperature used in the HIP process of the AgZ 1-3 sample. Similar secondary phases of Al and K (Figure 3d,e, respectively) were observed within the widespread Si matrix (Figure 3f). A different type of inclusion was observed in this image being comprised of Fe and Mn, the origin of these species is not known. (EDS not shown). Two common microstructural features are highlighted in the SEM micrograph (Figure 3a). Area #2 was measured to be comprised of higher levels of Na (3.4 wt %), Al (10.9 wt %), and K (5.1 wt %) with lower Ag (1.8 wt %) and I (0.2 wt %) compared to the bulk composition (see Table 3). Area #3 contained higher amounts of Ca (3.4 wt %) and Al (8 wt %) than the bulk.

**Figure 4:**
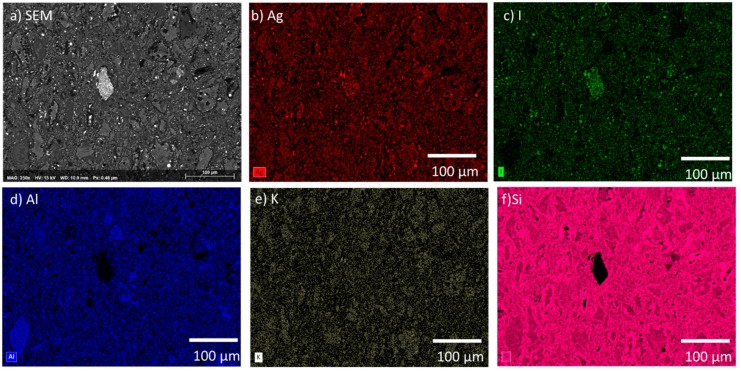
Microstructure of the AgZ 1-8 sample shown by by: (**a**) SEM micrograph; and the corresponding EDS maps of: (**b**) Ag; (**c**) I; (**d**) Al; (**e**) K; and (**f**) Si. The large white inclusion in (**a**) is Fe (source not known) with AgI particles within the structure.

Figure 4 displays the microstructure of the AgZ 1-8 sample. The elemental distributions across the microstructure were similar to AgZ 1-3 including small isolations of Ag that were associated with I in the AgZ 1-8. Such a distribution can be expected as the AgZ 1-3 and 1-8 sample had identical processing parameters. 

**Figure 5:**
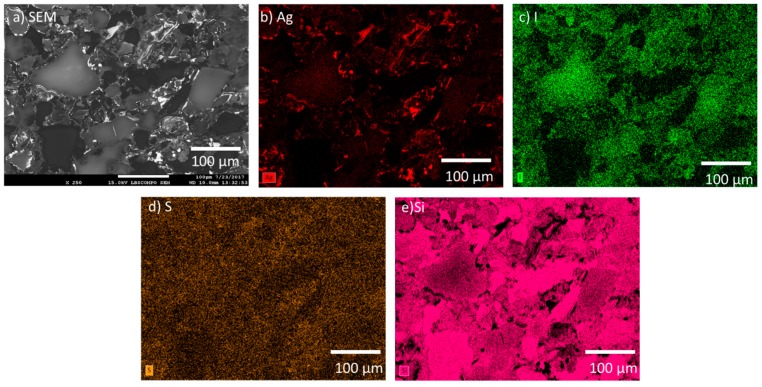
Microstructure of the SPS-1 sample shown by: (**a**) SEM micrograph; and the corresponding EDS maps of: (**b**) Ag; (**c**) I; (**d**) S; and (**e**) Si.

**Figure 6:**
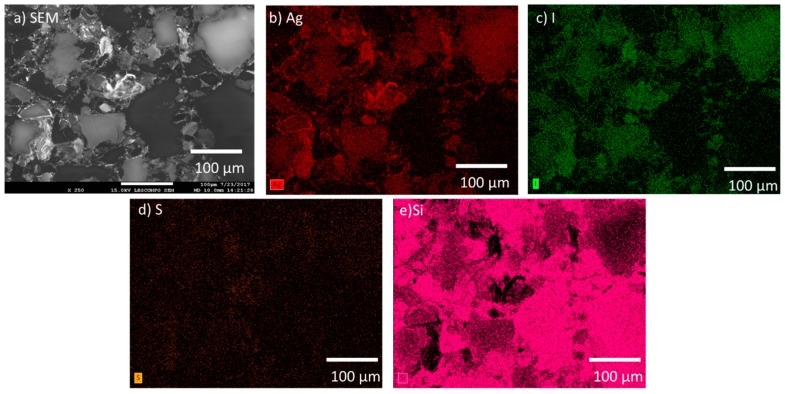
Microstructure of the SFA SPS-2 sample shown by: (**a**) SEM micrograph; and the corresponding EDS maps of: (**b**) Ag; (**c**) I; (**d**) S; and (**e**) Si.

The SFA samples were also comprised of a multiphase microstructure. The SPS-1 sample can be seen in the SEM micrograph in Figure 5a and large features were observed in the image. The Ag was observed to be sitting on the edges of the large particles and in smaller discrete isolations (see Figure 5b). The I was generally located throughout the sample but not as intimately associated with areas of high Ag (see Figure 5c). The SFA samples contained S from the thiol backbone of the original aerogel and the S was distributed evenly (Figure 5d), except for areas of high Si observed in Figure 5e. The SPS-2 sample, with 20 wt % additional Si added had a similar microstructure to the SPS-1 with a larger coverage of Si-rich particles (see Figure 6) and a more widespread distribution of Ag compared with the SPS-1 sample.

### 3.2. Corrosion Testing of HIPed Ag Mordenite

SPFT testing was performed on the AgZ samples with inlet solutions at pH 7, pH 9, and pH 11. The errors presented represent the standard deviation of the individual rates measured during the test. All dissolution rates in this work were normalized to the individual sample compositions (Table 1 and Table 2) and the dissolution rates of the samples were determined using the following equation:
$rate=\;\frac{\left[X\right]*V}{SA*t*f_{i}}$where
*X* is theconcentration of the analyte in the effluent, g/L;*V* is thevolume of the collected effluent during the interval, L;*SA* is thesurface area of the sample, m^2^;*t* is theduration of the interval, day; and*f*_i_ is thenormalization factor based on the mass % of analyte, unitless.

**Figure 7:**
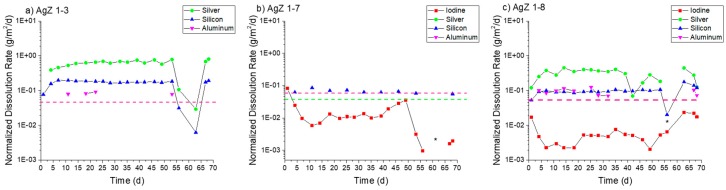
Normalized dissolution rates calculated from SPFT experiments using the AgZ samples at pH 7 for: (**a**) AgZ 1-3 (iodine-free sample); (**b**) AgZ 1-7; and (**c**) AgZ 1-8. The dashed lines, when present, represent the maximum rate for samplings where the analyte concentration was below the detection limit of the instrument and a dissolution rate calculated using the detection limit value. The asterisks (*) mark samplings where the flow rate deviated by >10% from the average flow of the test.

Figure 7 displays the normalized dissolution rates measured for the three AgZ samples in pH 7 solution. For the iodine-free AgZ 1-3 sample (Figure 7a), the dissolution rates were fairly constant over the duration of the test. The decreases observed at 63 days were due to a pump failure. The Ag dissolution rate was higher (0.65 ± 0.07 g/m^2^/day average) compared with the Si dissolution rate (0.17 ± 0.01 g/m^2^/day average). There was only detectable Al in four samples throughout the duration of the test, the rest falling below the instrument detection limit. Using the instrument detection limit for Al as an input, a maximum rate of 0.06 g/m^2^/day can be presumed for the Al dissolution rate. The AgZ 1-7 sample (Figure 7b) showed an average Si dissolution rate of 0.066 ± 0.009 g/m^2^/day while the I dissolution rate was lower at 0.015 ± 0.008 g/m^2^/day. Neither the Ag nor the Al had measurable concentrations in the effluent and maximum rates of 0.04 g/m^2^/day and 0.07 g/m^2^/day, respectively, can be assumed using the associated instrument detection limit. The AgZ 1-8 sample, having identical processing parameters to the AgZ 1-3 but with I (Figure 7c), behaved similarly to the iodine-free sample. The Ag dissolution rate was again higher (0.30 ± 0.11 g/m^2^/day) than the Si dissolution rate (0.08 ± 0.01 g/m^2^/day). The Al was measurable for this sample for the majority of the test with an average dissolution rate of 0.09 ± 0.02 g/m^2^/day being measured. The I dissolution rate was measured at 0.005 ± 0.001 g/m^2^/day, which was lower than the AgZ 1-7. Near the conclusion of the test, the I dissolution rate increased with time and is possibly due to the corrosion of the surface exposing more AgI that could dissolve. The difference between the AgZ 1-3 and Ag 1-8 Ag and Si dissolution rates compared with the AgZ 1-7 sample may have arisen from Ag particles being present outside of the Si matrix in the 1-3 and 1-8 samples (Figure 2 and Figure 4) and thus more readily attacked.

**Figure 8:**
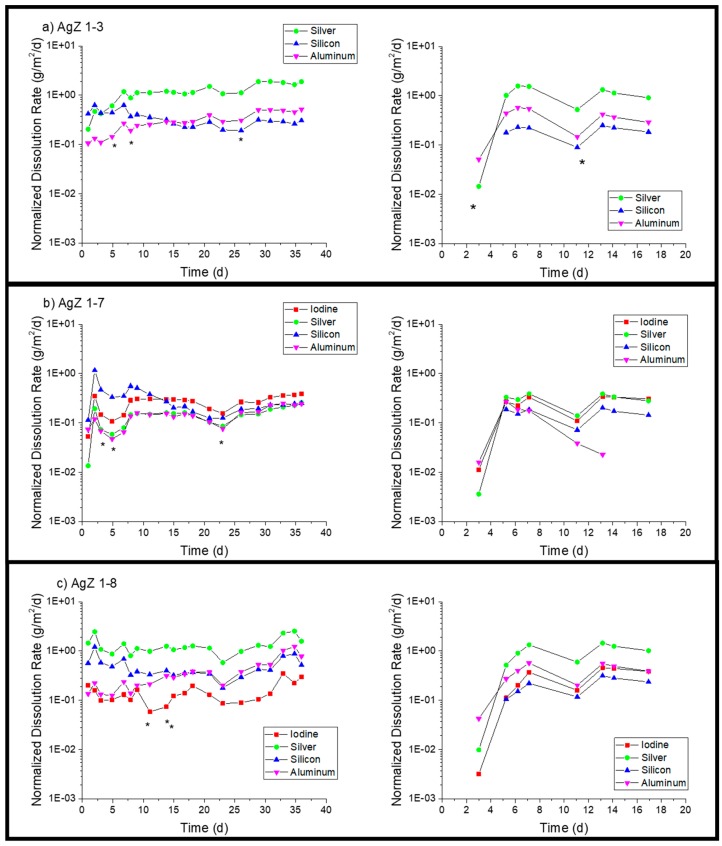
Normalized dissolution rates calculated from SPFT experiments using the AgZ samples at pH 9 for: (**a**) AgZ 1-3 (iodine-free sample); (**b**) AgZ 1-7; and (**c**) AgZ 1-8. The asterisks (*) mark samplings where the flow rate deviated by >10% from the average flow of the test.

Figure 8 presents the AgZ normalized dissolution rates in pH 9 solution. With the increased alkalinity of the test solution measurable analyte concentrates were present in all effluent samples. AgZ 1-3 (Figure 8a) showed a higher Ag dissolution rate (1.16 ± 0.49 g/m^2^/day in the 36-day test and 1.01 ± 0.37 g/m^2^/day in the 17-day test) compared with the Si dissolution rate (0.34 ± 0.12 g/m^2^/day in the 36-day test and 0.19 ± 0.05 g/m^2^/day in the 17-day test). The Al dissolution rates were measured to be 0.30 ± 0.13 g/m^2^/day (36 day) and 0.30 ± 0.13 g/m^2^/day (17 day). The AgZ 1-7 (Figure 8b) showed I dissolution rates of 0.25 ± 0.09 g/m^2^/day (36 day) and 0.27 ± 0.08 g/m^2^/day (17 day), Ag dissolution rates of 0.14 ± 0.05 g/m^2^/day (36 day) and 0.31 ± 0.08 g/m^2^/day (17 day), Si dissolution rates of 0.31 ± 0.23 g/m^2^/day (36 day) and 0.15 ± 0.04 g/m^2^/day (17 day) and Al dissolution rates of 0.14 ± 0.06 g/m^2^/day (36 day) and 0.13 ± 0.10 g/m^2^/day (17 day). The AgZ 1-8 (Figure 8c) showed I dissolution rates of 0.14 ± 0.06 g/m^2^/day (36-day test) and 0.30 ± 0.14 g/m^2^/day (17-day test), Ag dissolution rates of 1.32 ± 0.53 g/m^2^/day (36 day) and 1.01 ± 0.36 g/m^2^/day (17 day), Si dissolution rates of 0.49 ± 0.24 g/m^2^/day (36 day) and 0.20 ± 0.08 g/m^2^/day (17 day) and Al dissolution rates of 0.39 ± 0.30 g/m^2^/day (36 day) and 0.41 ± 0.13 g/m^2^/day (17 day). The measured dissolution rates at pH 9 in the 36-day tests and 17-day tests highlight the reproducibility using the SPFT technique. Similar to the pH 7 tests, the AgZ 1-3 and AgZ 1-8 samples showed similar dissolution rates with the rates for Ag being larger than the Si and Al. The AgZ 1-7 sample showed dissolution rates that tracked with one another for all four analytes. The I dissolution rates for AgZ 1-7 and AgZ 1-8 were similar despite the higher Ag dissolution rate for the AgZ 1-8. This would suggest some free Ag is generated at the higher HIP temperature of the AgZ 1-8 sample.

**Figure 9:**
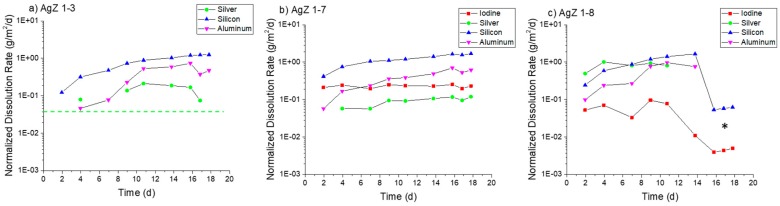
Normalized dissolution rates calculated from SPFT experiments using the AgZ samples at pH 11 for: (**a**) AgZ 1-3 (iodine-free sample); (**b**) AgZ 1-7; and (**c**) AgZ 1-8. The dashed lines, when present, represent the maximum rate for samplings where the analyte concentration was below the detection limit of the instrument and a dissolution rate calculated using the detection limit value. The asterisks (*) mark samplings where the flow rate deviated by >10% from the average flow of the test.

Moving to pH 11 (see Figure 9), an expected increase in overall dissolution of the samples was observed with new trends in the elemental releases. The AgZ 1-3 (Figure 9a) displayed an increase in dissolution rate until >7 days and the values measured beyond this were used to determine the average rates. The AgZ 1-3 sample showed higher Si dissolution rates (1.05 ± 0.21 g/m^2^/day) than Ag (0.14 ± 0.05 g/m^2^/day), which were different than rates for pH 7 and pH 9. The inversion of the two rates may have been due to the increased solubility of Si and potential decrease in Ag solubility (through formation of Ag_2_O) with increased alkalinity [31]. The Al dissolution rate was measured to be 0.49 ± 0.18 g/m^2^/day. The AgZ 1-7 (Figure 9b) showed a similar trend with a Si dissolution rate of 1.20 ± 0.32 g/m^2^/day and an Ag dissolution rate of 0.09 ± 0.02 g/m^2^/day. The I dissolution rate was measured to be 0.22 ± 0.02 g/m^2^/day and the Al dissolution rate was 0.39 ± 0.18 g/m^2^/day. AgZ 1-8 (Figure 9c) showed a Si dissolution rate of 0.99 ± 0.46 g/m^2^/day, an Ag dissolution rate of 0.81 ± 0.19 g/m^2^/day, an I dissolution rate of 0.06 ± 0.02 g/m^2^/day, and an Al dissolution rate of 0.52 ± 0.38 g/m^2^/day. The last three sampling of the pH 11 test had a lower flow rate through the reactor and conditions within the reactor may have changed, leading to the stark decreases observed after 14 days. 

**Figure 10:**
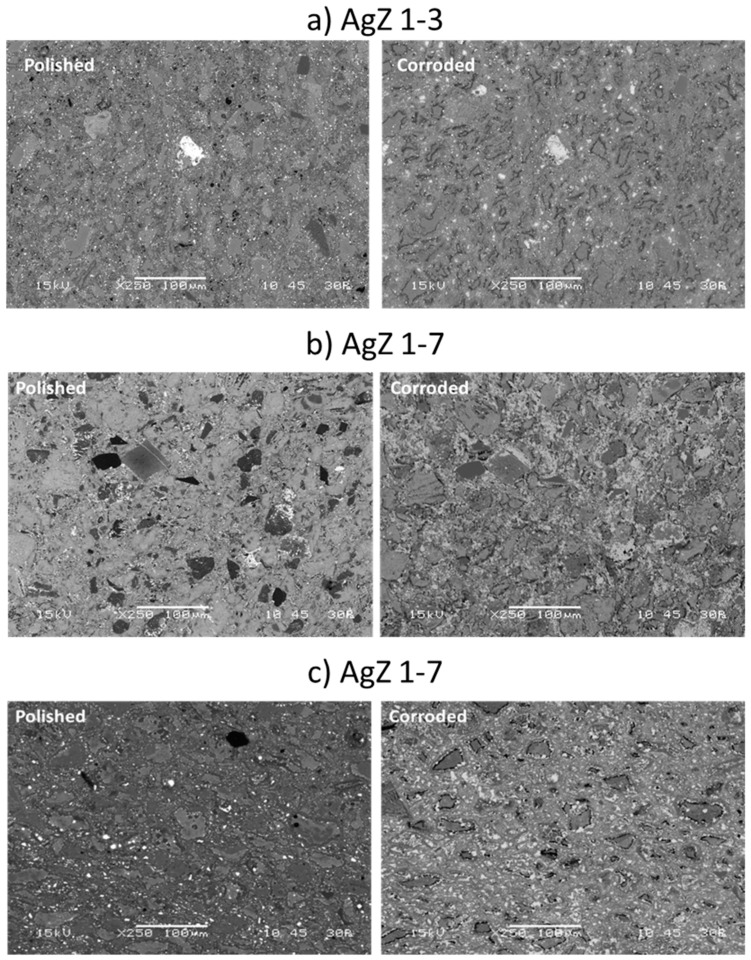
SEM micrographs of the samples both before and after corrosion in pH 11 SPFT experiments: (**a**) AgZ 1-3; (**b**) AgZ 1-7; and (**c**) AgZ 1-8.

Based on the solution data presented above, an incongruent dissolution of the sample surface is likely occurring. The different phases of the heterogeneous microstructure shown in Figure 1, Figure 2 and Figure 3 can each corrode independently of one another. The monolithic samples were imaged following SPFT testing to observe any physical changes on the sample surface. Using SEM, no observable changes were present on the AgZ samples following the pH 7 and pH 9 tests. Following the pH 11 tests, noticeable changes were present on the AgZ samples. Figure 10 shows the AgZ sample surfaces before and after the pH 11 exposure. The AgZ 1-3 sample (Figure 10a) appears to have corroded at the secondary phases and not the continuous Si matrix. The Ag particles (bright spots) appeared larger following corrosion as the higher alkalinity environment may increase their stability while the rest of the material corrodes. Based on the Pourbaix diagram for Ag, above pH 9 AgO becomes a stable phase for Ag and such a process may be occurring in the pH 11 tests [32]. For the AgZ 1-7 sample (Figure 10b), the secondary phases also appeared to have corroded. This observation is best exemplified by the rhomboid-shaped particle in the left center of the image, which was a K-rich particle. Following corrosion, the sharp edges of this phase had disappeared. The AgZ 1-8 sample also showed attack of the secondary phases and, similar to the AgZ 1-3 sample, an apparent growth of the Ag-containing particles (Figure 10c). The SEM micrographs suggest that corrosion preferentially occurred at the secondary phases, yet this was only observed from a two-dimensional view.

**Figure 11:**
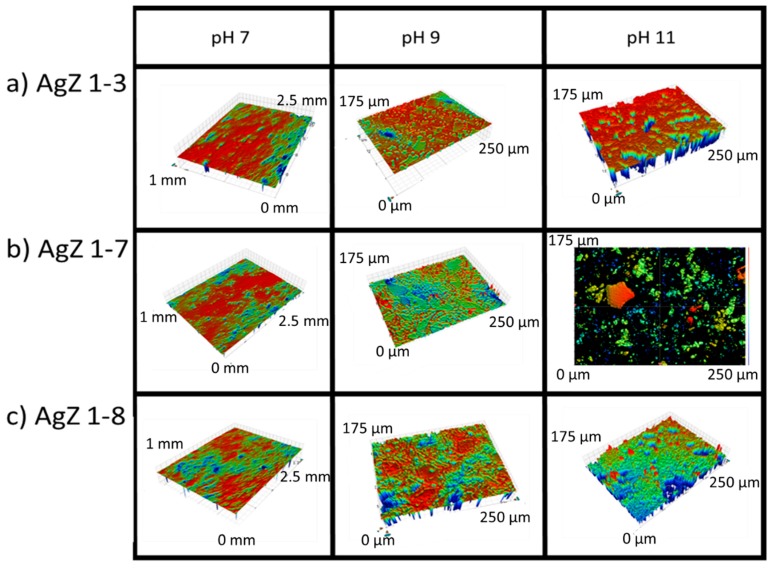
Optical profilometry images of the samples following the SPFT experiments: (**a**) AgZ 1-3; (**b**) AgZ 1-7; and (**c**) AgZ 1-8. At pH 7, the surface could not be resolved at the higher magnification (50×) so a lower magnification (5×) was used.

Optical profilometry was used to observe the three-dimensional (3D) profile of the AgZ samples following the SPFT experiments (Figure 11). At pH 7, all three samples showed only minor surface topography. In fact, the surface had retained enough of its polished nature to make it difficult to create the proper reflection to image at higher resolution, and as a result, a lower magnification image is shown. At pH 9, the surface morphology resembled what was suggested by the SEM images in Figure 10. Here, the lowest points on the surface were found to be the secondary phases for all three samples. The shapes and distributions of the phases suggest that these are the alkali- and alkaline-earth-rich phases shown in Figure 2, Figure 3 and Figure 4. At pH 11, more extensive damage was observed and the AgZ 1-7 sample could not be fully resolved to generate a 3D image. 

XRD analysis of the AgZ samples following testing at pH 9 and pH 11 showed no substantial difference (spectra not shown) to the starting material [26]. Following the test, the sample surface was composed of a mixture of silicon oxides, Ag metal, and AgI (Table 4). It should be noted that the XRD mode used generated excitation volumes between 5 µm and 50 µm and the information within this table includes the signal from the surface and inner sample in the excitation volume.

### 3.3. Corrosion Testing of Spark Plasma Sintered Silver-Functionalized Silica Aerogels

**Figure 12:**
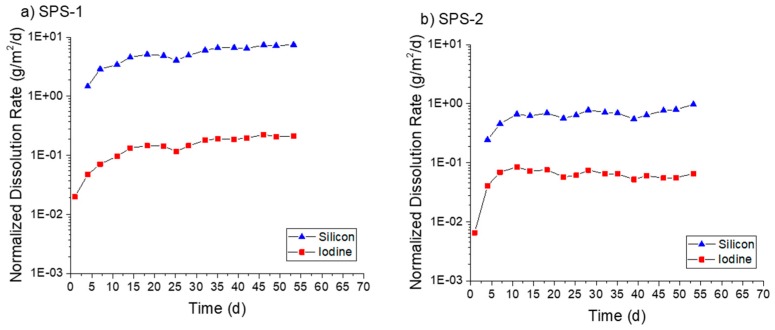
Normalized dissolution rates calculated from SPFT experiments using the SFA samples at pH 7 for: (**a**) SPS-1; and (**b**) SPS-2 (+20 wt % SFA). The asterisks (*) mark samplings where the flow rate deviated by >10% from the average flow of the test.

The densified SFA materials were tested using the SPFT method in a similar fashion to the AgZ. In pH 7 solution (Figure 12a), the SPS-1 sample experienced consistent dissolution, with an average Si dissolution rate of 4.49 ± 1.52 g/m^2^/day and an I dissolution rate of 0.12 ± 0.05 g/m^2^/day. The SPS-2 sample (Figure 12b), with higher Si content, measured lower Si dissolution rates averaging 0.65 ± 0.16 g/m^2^/day and I dissolution rates measuring 0.06 ± 0.02 g/m^2^/day. No Ag release was measured at pH 7 for either sample. 

**Figure 13:**
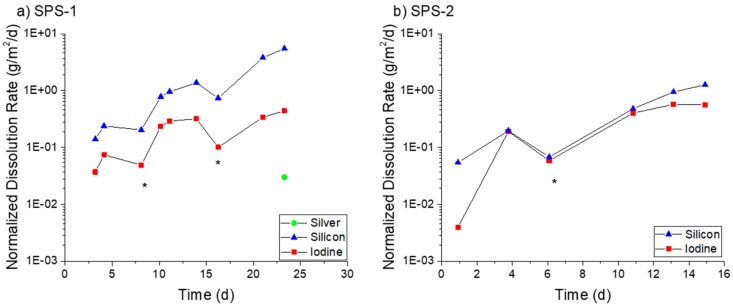
Normalized dissolution rates calculated from SPFT experiments using the SFA samples at pH 9 for: (**a**) SPS-1; and (**b**) SPS-2 (+20 wt % SFA). The asterisks (*) mark samplings where the flow rate deviated by >10% from the average flow of the test.

In pH 9 solution (Figure 13), both samples showed a continual increase in dissolution rate with time. At the conclusion of the test on the SPS-1 sample (Figure 13a), the measured Si dissolution rate was 4.67 g/m^2^/day and the measured I dissolution rate was 0.37 g/m^2^/day. The SPS-2 sample (Figure 13b) showed a Si dissolution rate of 1.26 g/m^2^/day and an I dissolution rate of 0.56 g/m^2^/day. Only at the conclusion of the test was Ag measurable for the SPS-1 sample, corresponding to an Ag dissolution rate of 0.02 g/m^2^/day. No Ag release was measurable for the SPS-2 sample.

**Figure 14:**
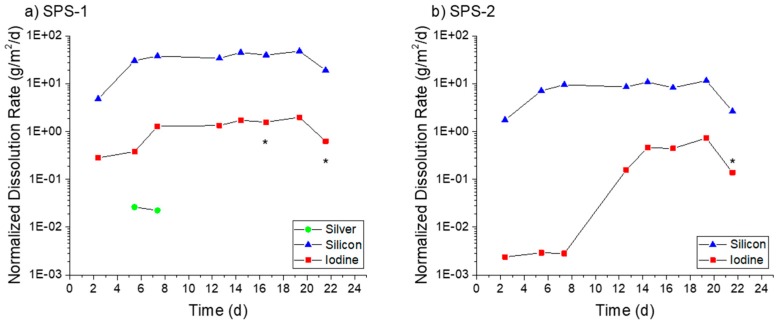
Normalized dissolution rates calculated from SPFT experiments using the SFA samples at pH 11 for: (**a**) SPS-1; and (**b**) SPS-2 (+20 wt % SFA). The asterisks (*) mark samplings where the flow rate deviated by >10% from the average flow of the test.

At pH 11 (Figure 14a), the SPS-1 sample measured an average Si dissolution rate of 33.3 ± 5.6 g/m^2^/day and an I dissolution rate of 1.04 ± 0.56 g/m^2^/day prior to the decrease at the final interval. The SPS-2 sample (Figure 14b) showed a Si dissolution rate of 10.21 ± 1.73 g/m^2^/day. The I release was initially low before increasing past seven days. After this increase, the average I dissolution was measured at 0.54 ± 0.16 g/m^2^/day. Ag was measured in the effluent at two time points for the SPS-1 sample equaling an Ag dissolution rate of 0.02 g/m^2^/day.

In all cases, the Si dissolution rates measured for SPS-2 were lower than those for SPS-1. The SPS-2 sample had additional Si added (as raw SFA) prior to sintering to improve durability and this methodology appeared to be successful. With the exception of the SPS-2 at pH 9, the I dissolution rates were also lower than the Si dissolution rates for the SFA samples. The microstructure of the SFA samples had areas of higher Si without any I present. Dissolution of those particles may have caused the higher Si dissolution rates, and more-so if the Si isolations were less durable than the matrix. The minimal release of Ag observed may be a result of the S presence in the SFA. AgS is a very insoluble compound and it is possible that dissolved Ag can become associated with S and be retained on the surface.

**Figure 15:**
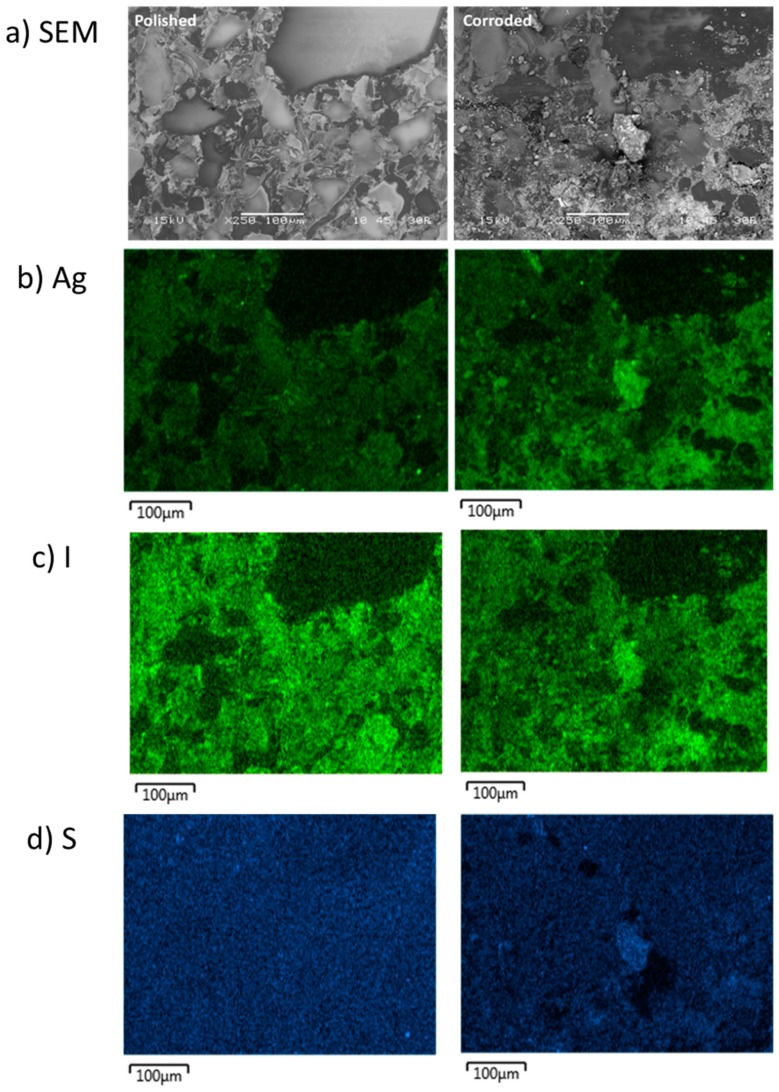
(**a**) SEM micrographs of the SPS-1 sample (left) before and (right) following SPFT testing at pH 11; and the corresponding EDS maps of: (**b**) Ag; (**c**) I; and (**d**) S.

As with the AgZ samples, there were observable changes on the SFA surfaces following pH 11 exposure. SEM-EDS analysis performed on SPS-1, shown in Figure 15, provided some insight as to the retention of Ag during the testing of the SFA samples. In the SEM micrographs (Figure 15a), the large Si particles in the uncorroded image (those depleted in Ag and I) were heavily corroded, the large particle in the upper right being a perfect example. The attack appeared to have moved from the outer edge of the particles inward. The Ag remained evenly distributed following corrosion while some new Ag particles also appeared (Figure 15b). The I-rich particles in the uncorroded image near the large particles had disappeared in the corroded image (Figure 15c). The dissolution of the large particles likely drove the I-release. The most prominent change, however, is the increased definition of S in the image following corrosion (Figure 15d). After corrosion, a large particle has been exposed or generated that also contained Ag (Figure 15b) and I (Figure 15c). The appearance of the Ag-S-containing particles coordinated with I (see the large particle in the center of the corroded images) suggests that S may be responsible for the low Ag release and present a possible mechanism for improving I-retention in the sintered SFA. Previous work has shown that S behaves as a redox control agent over the Ag [33]. More work is planned to pursue understanding of this possible mechanism.

**Figure 16:**
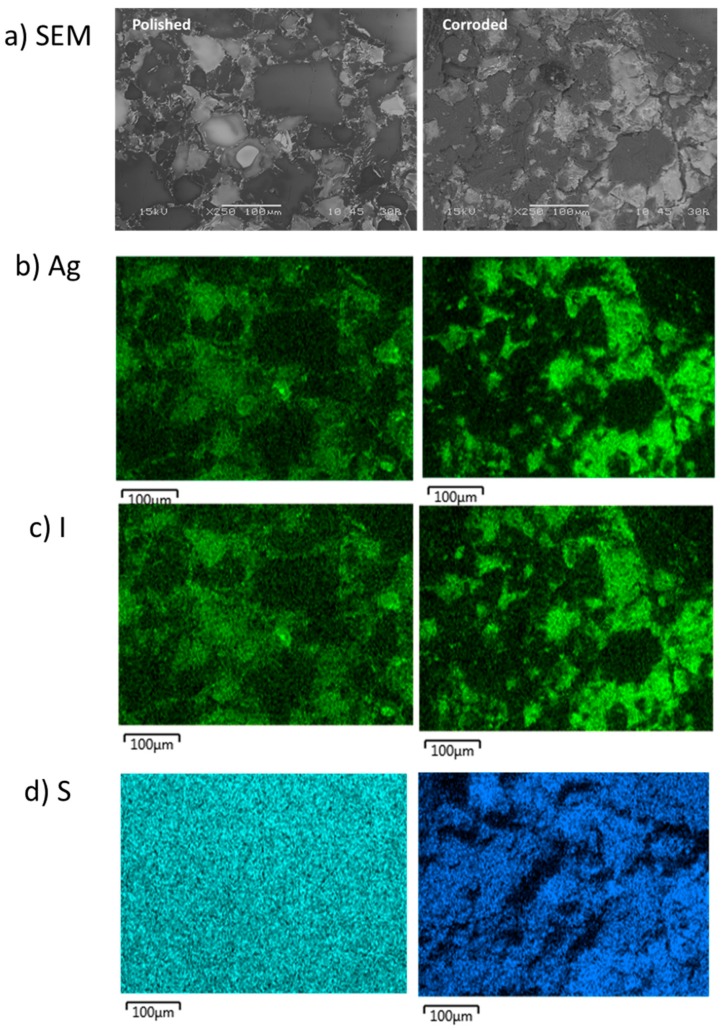
(**a**) SEM micrographs of the SPS-2 sample before (left) and following (right) SPFT testing at pH 11; and the corresponding EDS maps of: (**b**) Ag; (**c**) I; and (**d**) S.

For the SPS-2 sample, clear corrosion attack of the Si-rich particles was observed (Figure 16a), with the large particle in the center of the image being almost fully removed. An AgI particle was observed in the center of the non-corroded image (Figure 16b,c). Following corrosion, this particle was more visible as a result of the Si matrix removal around the particle. Other large AgI isolations behaved similarly in the images. Compared with the small AgI particles at the boundaries of the Si-particles in SPS-1 that were removed, large AgI isolations appeared to be retained better on the surface of the SPS-2.

**Figure 17:**
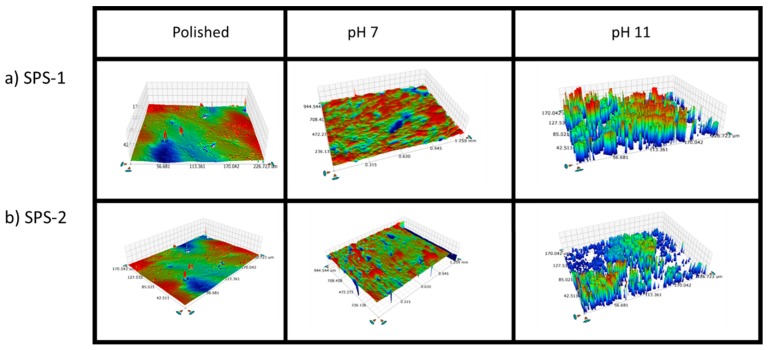
Optical profilometry images before and after the SPFT experiments at pH 7 and pH 11 of: (**a**) SPS-1; and (**b**) SPS-2.

The change in overall surface roughness of the SFA samples based on exposure pH can be observed in the optical profilometry images shown in Figure 17. For both the SPS-1 (Figure 17a) and SPS-2 (Figure 17b), following exposure at pH 7, the surface was notably roughened compared with the polished surface. The suppressed regions of the sample following corrosion appeared to be isolated and would suggest a similar dissolution pathway targeting the Si particles, as was observed at pH 11 in the SEM images. The surface was heavily corroded at pH 11.

### 3.4. Comparison to Other Materials

Dissolution rates for Si-based materials in SPFT testing are highly dependent on the flow to surface area (*q*/*S*) ratio. This can limit direct comparisons between the dissolution rates measured for materials in different SPFT testing efforts. However, any comparative assessment of the overall durability of IWFs should be made against other material types under investigation for the long-term disposal of nuclear wastes. Other iodine-containing waste forms have been tested with SPFT but the tests were performed with differing conditions. Neeway et al. performed SPFT on iodine-containing fluidized bed steam reforming (FBSR) material at 40 °C and at far lower *q*/*S* (largest being 3 × 10^−4^ m/day) than this work [34]. Higher temperature dissolution data on FBSR material (without iodine) has been reported but the *q*/*S* used in the testing was not included [35]. Mowry et al. used a small-volume SPFT design to assess the durability of low-temperature Bi-Si-Zn oxide glass-composite materials (GCM) that contained AgZ [36]. The experiments focused on solutions with pH < 7, a maximum temperature of 60 °C and a *q*/*S* of 2 × 10^−4^ m/day. An iodine-containing glass (BNDL-A-S98) was investigated with SPFT at 90 °C but the raw data is not available in the report to compare the *q*/*S* values [37]. The best available comparisons are works on the dissolution of high-level nuclear waste glasses [38] and glass ceramic waste forms [39] where SPFT tests were performed (on powdered samples) at 90 °C in pH 9 and pH 11 solutions with similar *q*/*S* values to this work. The glass ceramic waste forms were multi-phase, borosilicate-based materials comprised of a borosilicate glass matrix with crystalline powellite and oxyapatite phases within. A summary of the normalized dissolution rates determined in this study as well as the comparative examples is given in Table 5. The three high-level waste glasses AFCI, ISG, and SON68 had Si dissolution rates of 0.350 g/m^2^/day, 0.154 g/m^2^/day, and 0.369 g/m^2^/day, respectively, while the glass-ceramic waste form had a Si dissolution rate of 3.39 g/m^2^/day in pH 9 tests. The highest pH 9 dissolution rates in the current study were 0.20 g/m^2^/day for AgZ 1-8 and 4.67 g/m^2^/day for SPS-1. This comparison suggests that the Si-matrices of the IWFs in the study are as durable as other Si-based waste form materials at pH 9. At pH 11, ISG had the highest Si dissolution rate at 3.44 g/m^2^/day while AgZ 1-7 had a Si dissolution rate of 1.20 g/m^2^/day and SPS-1 was higher at 33.3 g/m^2^/day. The limited number of available datasets to directly compare IWF durability highlights the need for a standardized test to be defined to assess IWFs on an even playing field and to provide data to be used in long-term modelling predictions of IWF durability upon disposal.

## 4. Conclusions

In summary, the dilute-condition chemical durability of two IWF types, HIPed AgZ and SPS-SFA, were investigated using the SPFT method. For the AgZ samples, the following trends were observed: (1) at pH 7 and 9, the releases of Ag were larger than that of Si for samples HIPed at higher temperature; (2) at pH 7, the release of I was much slower compared to the other analytes; (3) at pH 11, the release of Si was higher than Ag; and (4) preferential corrosion attack was observed on secondary phases that contained higher amounts of Al and alkali species but were lower in overall I content. The following observations were made for the SFA samples: (1) lowered I release compared with Si; (2) corrosion attack was preferential at Si-rich particles making small AgI isolations near these particle boundaries susceptible to dissolution; (3) an increased addition of Si (in the form of 20 wt % SFA) to the SFA waste form improved chemical durability; (4) minimal Ag release was observed; and (5) Ag- and S-containing isolations were found to contain I after corrosion testing. Both IWF types had similar dissolution rates to other Si-based waste forms at pH 9 and pH 11. The information collected here can be used in the development of long-term predictive models for disposal of IWFs, help direct improved chemical durability of the IWFs, and highlighted a need for a standardized test to be used for the durability of IWF.

## Figures and Tables

**Table 1:** Composition of the AgZ samples used in this study determined using the average area scan from multiple EDS maps. The standard deviation is representative of the multiple areas imaged to determine the average composition.

Sample	AgZ 1-3		AgZ 1-7		AgZ 1-8	
Element	wt %	St.Dev	wt %	St.Dev	wt %	St.Dev
Ag	8.70	2.21	10.94	1.74	9.78	1.57
I	0.00	0.00	6.02	1.34	4.79	1.52
O	43.10	2.02	37.84	2.96	39.85	2.23
Na	0.30	0.10	0.27	0.19	0.22	0.21
Mg	0.62	0.11	0.53	0.11	0.38	0.24
Al	6.48	0.75	6.18	0.90	6.02	0.59
Si	34.64	2.89	33.73	3.59	33.85	3.07
K	0.66	0.17	0.70	0.25	0.53	0.24
Ca	0.87	0.08	0.86	0.15	0.74	0.23
Fe	1.20	0.86	1.35	1.05	1.45	0.96
Others	3.42	-	1.57	-	2.39	-

**Table 2:** Composition of the SFA SPS samples based on the initial composition of the SFA material. The “Others” is comprised primarily of oxygen and minor species (e.g., Fe).

Sample	SPS-1	SPS-2
Element	wt %	wt %
Ag	24.9	19.9
I	30.0	24.0
Si	16.8	33.0
S	0.7	0.5
Others	27.6	22.1

**Table 3:** Composition of the features highlighted in the SEM images in Figure 2a and Figure 3a determined with EDS spot analysis.

Image	Figure 2a	Figure 3a	Figure 3a
Location	1	2	3
Element	wt %	wt %	wt %
**Ag**	5.7	1.8	4.5
**I**	0	0.2	0.4
**O**	46.3	41.6	43.0
**Na**	0.5	3.4	0.2
**Mg**	0.3	0.08	0.9
**Al**	6.9	10.9	8.0
**Si**	37.2	35.5	36.9
**K**	1.5	5.1	0.9
**Ca**	0.6	0.2	3.4
**Fe**	0.3	0.3	0.2
**Others**	0.7	0.92	1.6

**Table 4:** Summary of the crystalline phases measured with XRD following SPFT AgZ tests at pH 9 and pH 11.

Sample	pH 9	pH 11
**AgZ 1-3**	Ag metal, aluminum silicon oxide, silicon oxide, anorthite	Ag metal, aluminum silicon oxide, silicon oxide
**AgZ 1-7**	Ag metal, silicone oxide, anorthite, Ag iodide, aluminum silicate, cristobalite	Ag metal, silicone oxide, anorthite, Ag iodide, aluminum silicate, cristobalite
**AgZ 1-8**	Ag metal, silicone oxide, anorthite, Ag iodide, aluminum silicate, cristobalite	Ag metal, silicone oxide, anorthite, Ag iodide, aluminum silicate, cristobalite

**Table 5:** Summary of the normalized dissolution rates measured with SPFT testing in this report and comparison to dissolution rates measured for other materials in similar test conditions.

Sample	Test pH (Room Temp)	Length (d)	*q*/*S* (m/day)	I Dissolution Rate (g/m^2^/day)	Ag Dissolution Rate (g/m^2^/day)	Si Dissolution Rate (g/m^2^/day)	Al Dissolution Rate (g/m^2^/day)
AgZ 1-3	7	68	0.21	*N/A*	0.65 ± 0.07	0.17 ± 0.01	*< 0.06*
	9	17	0.18	*N/A*	1.01 ± 0.37	0.19 ± 0.05	0.35 ± 0.14
		36	0.17	*N/A*	1.16 ± 0.49	0.34 ± 0.12	0.30 ± 0.13
	11	18	0.21	*N/A*	0.14 ± 0.05	1.05 ± 0.21	0.49 ± 0.18
AgZ 1-7	7	68	0.24	0.015 ± 0.008	*<0.04*	0.066 ± 0.009	*< 0.07*
	9	17	0.21	0.27 ± 0.08	0.31 ± 0.08	0.15 ± 0.04	0.13 ± 0.10
		36	0.17	0.25 ± 0.09	0.14 ± 0.06	0.32 ± 0.23	0.14 ± 0.06
	11	18	0.23	0.22 ± 0.02	0.09 ± 0.02	1.20 ± 0.32	0.39 ± 0.18
AgZ 1-8	7	68	0.24	0.005 ± 0.001	0.30 ± 0.11	0.08 ± 0.01	0.09 ± 0.02
	9	17	0.18	0.30 ± 0.14	1.01 ± 0.36	0.20 ± 0.08	0.41 ± 0.13
		36	0.17	0.14 ± 0.07	1.32 ± 0.53	0.49 ± 0.24	0.39 ± 0.30
	11	18	0.23	0.06 ± 0.02	0.81 ± 0.19	0.99 ± 0.46	0.52 ± 0.38
SPS-1	7	68	0.41	0.12 ± 0.05	ND	4.49 ± 1.52	NA
	9	17	0.36	0.37	0.02	4.67	NA
	11	18	0.31	1.04 ± 0.56	0.02	33.3 ± 5.6	NA
SPS-2	7	68	0.37	0.06 ± 0.02	ND	0.65 ± 0.16	NA
	9	17	0.32	0.56	ND	1.26	NA
	11	18	0.28	0.54 ± 0.16	ND	10.21 ± 1.73	NA
AFCI (€)	9	21	0.35	NA	NA	0.350	NA
	11	21	0.35	NA	NA	3.36	NA
ISG (€)	9	21	0.35	NA	NA	0.154	NA
	11	21	0.35	NA	NA	3.44	NA
SON68 (€)	9	21	0.35	NA	NA	0.369	NA
	11	21	0.35	NA	NA	2.11	NA
Glass Ceramic (¥)	9	21	4.1E-01	NA	NA	3.39	NA

NA, data not available or analyte not present in sample; ND, analyte below detection limit, thus no rate calculations; € Ref [38]; ¥, Ref [39].
